# Secreted Factors and Extracellular Vesicles Account for the Immunomodulatory and Tissue Regenerative Properties of Bone-Marrow-Derived Mesenchymal Stromal Cells for Osteoarthritis

**DOI:** 10.3390/cells11213501

**Published:** 2022-11-04

**Authors:** Enrico Ragni, Carlotta Perucca Orfei, Laura de Girolamo

**Affiliations:** Laboratorio di Biotecnologie Applicate all’Ortopedia, IRCCS Istituto Ortopedico Galeazzi, Via R. Galeazzi 4, I-20161 Milan, Italy

**Keywords:** bone marrow, mesenchymal stromal cells, secretome, extracellular vesicles, miRNA, osteoarthritis

## Abstract

Bone-marrow-derived mesenchymal stromal cells (BMSCs) showed therapeutic potential in the treatment of musculoskeletal diseases, including osteoarthritis (OA). Their soluble mediators and extracellular vesicles (EVs), which make up the secretome, suppress immune response, attenuate inflammation and promote cartilage repair. EVs, as well as the whole secretome, have been investigated as cell free approaches for OA although, to date, a disease-tailored molecular fingerprint is missing. In this study, soluble mediators and miRNAs were sifted in the BMSCs’ secretome and EVs, respectively, and analyzed in the frame of cell types and factors involved in OA. The majority of identified molecules repress the activation of immune cells and the production of OA-related inflammatory mediators, as well as promote cartilage protection by acting on both chondrocytes homeostasis and extracellular matrix-degrading enzymes. These data provide the molecular ground for the therapeutic potential of BMSCs for regenerative applications for OA and support the use of secretome or EVs as cell-free applications in joint diseases.

## 1. Introduction

Osteoarthritis (OA) is the most common degenerative joint disorder affecting more than 500 million people worldwide, with particular prevalence in those >65 years of age [1]. OA is characterized by changes across all joint tissues, in particular cartilage and synovial membrane [2]. Although often underestimated, synovitis is associated with cartilage damage [3] and fibroblast-like synoviocytes (FLS) proliferation [4], M1 inflammatory macrophage recruitment [4] and T cell activation [5]. No effective therapies are available to halt or delay OA progression, and joint replacement with an artificial prosthesis is still the most effective measure to improve patient quality of life [6]. For this reason, current clinical trials are mainly focused to restore a suitable microenvironment for cartilage regeneration/repair and targeting of pro-inflammatory cells/mediators by means of intra-articular injection of chemicals or biologics. Among orthobiologics, mesenchymal stromal cells (MSCs) gained interest due to their regenerative and anti-inflammatory properties, with particular attention on bone marrow as an MSC source (BMSCs) [7] due to its ease of harvest and lack of ethical issues, although sometimes it has lower availability with respect to other sources such as fat or placenta. For these reasons, to date, more than 30 clinical trials using BMSCs-based products have been registered under https://www.clinicaltrials.gov/ (accessed on 30 September 2022) for OA.

In the last year, it has become evident that the regenerative and immunomodulatory properties of MSCs relies on their capacity to secrete bioactive molecules [8]. The secreted molecules, either free or conveyed within extracellular vesicles (EVs), are collectively termed the “secretome”. With respect to the resolution of inflammation and promotion of cartilage regeneration, both the whole secretome and purified EVs have showed promising results in in vivo OA models [9]. Accordingly, in March 2020 and September 2021, the first clinical studies which explored the possibility of using clinical-grade MSC secretome or EVs for the treatment of OA were registered (https://www.clinicaltrials.gov/, NCT04314661 and NCT05060107, accessed on 30 September 2022). Nevertheless, the promising results of both BMSCs and their secretome/Evs in OA field [10,11] have not yet been associated to a thorough disease-related fingerprint of soluble molecules, including cytokine/chemokines or EV-miRNAs. This would be of particular relevance since it would help to better understand the therapeutic potential of BMSCs and their released factors/miRNAs in the frame of those reported to influence OA-related cell types and disease progression or resolution [12,13].

The aim of this work was to characterize the presence of soluble factors In the whole secretome and miRNAs in purified eVs from BMSCs. Identified molecules were analyzed using a disease-related approach. OA molecular determinants and OA-affected cell types and tissues, such as cartilage, FLS, macrophages and T cells, were all covered. The data obtained will provide a disease-focused molecular perspective of the therapeutic properties of BMSCs and their secretomes/EVs when used in the treatment of OA.

## 2. Materials and Methods

### 2.1. Bone Marrow Retrieval and BMSC Isolation and Expansion

Total bone marrow was collected from the iliac crest of 3 female OA patients (mean age: 50 ± 2, Kellgren and Lawrence II–III) and seeded at 50,000 total nucleated cells/cm^2^ in αMEM (Thermo Fisher Scientific, Waltham, MA, USA) supplemented with 10% FBS at 37 °C, 5% CO_2_ and 95% humidity. After 3 days, the supernatant was discarded and replaced by fresh complete medium. BMSCs were selected due to their plastic adhesion and, after 2 weeks, colonies were detached and BMSCs seeded at 4000 cells/cm^2^. For secretome collection, BMSCs at passage 3 and 90% confluence were washed 3 times with PBS, and serum-free αMEM was added at a ratio of 0.07 mL/cm^2^. After 48 h, conditioned medium was collected and centrifuged (376× *g* for 5 min at 4 °C, 1000× *g* for 15 min at 4 °C, 2000× *g* for 15 min at 4 °C and twice at 4000× *g* for 15 min at 4 °C), recovering the supernatant at each run to be further processed with the following centrifugation. Eventually, clarified secretome was split and used for analysis of EVs as well as enzyme-linked immunosorbent assay (ELISA). After secretome removal, BMSCs were detached and counted before being assessed for viability using a NucleoCounter NC-3000 (ChemoMetec, Allerod, Denmark).

### 2.2. BMSCs Characterization by Flow Cytometry

After 30 min of staining at 4 °C in the dark and antibody wash with FACS buffer (PBS, 5% FBS, 0.1% sodium azide), MSC (CD44-PE Vio770 clone REA690, CD73-PE clone REA804, CD90-FITC clone REA897, CD105-PerCP Vio700 clone REA794 and CD271-PE clone REA844) or hemato/endothelial (CD31-PerCP Vio700 clone REA730, CD34-FITC clone AC136 and CD45-PE Vio770 clone REA747) markers (Miltenyi Biotec, Bergisch Gladbach, Germany) were detected by flow cytometry using a CytoFLEX flow cytometer (Beckman Coulter, Fullerton, CA, USA). A minimum of 30,000 events were collected. The following antibody combinations were used: CD73/90/105/44 and CD34/271/31/45.

### 2.3. Multiplex ELISA Assay

Two-hundred soluble receptors, chemokines, cytokines, growth and inflammatory factors were quantified by Quantibody^®^ Human Cytokine Array 4000 Kit (https://www.raybiotech.com/quantibody-human-cytokine-array-4000/, accessed on 30 September 2022) in the cleared BMSCs secretome according to the manufacturers’ protocol (RayBiotech, Norcross, GA, USA). To allow the absorbance readings within the standard curve values, a 1:1 dilution was performed. For each presented value, the mean of 4 technical replicates is shown. The amount of each factor in pg/mL was converted into pg/million cells by multiplying the original value for the total collected volume in ml and finally dividing by the total number of cells. Values are shown as mean ± SD.

### 2.4. Protein-Protein Interaction Networks

The online tool STRING (http://www.string-db.org, accessed on 13 May 2022) was used to build interactome maps of ELISA-identified proteins (database v11.5) with the following properties: (i) organism, Homo Sapiens; (ii) meaning of network edges, evidence; (iii) active interaction sources, experiments; and (iv) minimum required interaction scores, low confidence (0.150).

### 2.5. EVs Characterization

Flow cytometry: Cleared secretome was 1:1 diluted with PBS and divided into 3 aliquots: (i) unstained, (ii) CFSE (1 µM final concentration) stained for 30 min at 37 °C and (iii) after CFSE supplementation, further staining for 30 min at 4 °C with one of the following antibodies: CD9-APC clone HI9A, CD63-APC clone H5C6, CD81-APC clone 5A6, CD44-APC clone BJ18, CD73-APC clone AD2 or CD90-APC clone 5E10) (Biolegend, San Die-go, CA, USA). After a further 1:3 dilution with PBS, samples were analyzed with a CytoFlex flow cytometer. FITC-fluorescent beads of 160, 200, 240 and 500 nm (Biocytex, Mar-seille, France) were used as an internal control. At least 30,000 events were collected.

Nanoparticle tracking analysis (NTA): Cleared secretome was 1:1 diluted in PBS and visualized by Nanosight LM10-HS system (NanoSight Ltd., Amesbury, UK). Each sample was run with 5 recordings of 60 s. NTA software provided both concentration measurements and high-resolution distribution profiles of particle size.

### 2.6. Total RNA Isolation and miRNA Quantification

Five ml of cleared secretome was 1:1 diluted in PBS before ultracentrifugation at 100,000× *g* for 9 h at 4 °C. Resulting pellets were processed with miRNeasy and RNeasy Cleanup Kits (Qiagen, Hilden, Germany) after addition of 30 pg of exogenous *Arabidopsis thaliana* ath-miR-159a synthetic miRNA spike. This was used to evaluate RNA recovery and cDNA synthesis performed as previously reported [14]. The OpenArray system (Life Technologies, Foster City, CA, USA) was used to determine miRNA expression in 384-well OpenArray plates according to the manufacturer’s protocol. Each single miRNA was considered as present when amplification resulted in all three samples. Eventually, ath-miR-159 spike-in C_RT_ was used to equalize technical differences, and the global mean method [15] allowed normalization between samples.

### 2.7. miRNA Target Identification

miRTarBase v8.0 (https://mirtarbase.cuhk.edu.cn/~miRTarBase/miRTarBase_2022/php/index.php, accessed on 14 March 2022) was used to analyze miRNAs under analysis to identify mRNA targets [16]. Only miRNA–mRNA interactions supported by strong experimental evidence were considered.

### 2.8. Statistical Analyses

GraphPad Prism Software version 5 (GraphPad, San Diego, CA, USA) was used to perform statistical analyses. Linear association between samples was determined by Pearson’s correlation coefficient (R^2^) and the outcome results were interpreted according to the degree of association [17].

ClustVis package (https://biit.cs.ut.ee/clustvis/, accessed on 13 May 2022) [18] was used to perform principal component analysis (PCA) and hierarchical clustering on normalized C_RT_ values. After row centering, maps were generated using the following settings for both rows’ and columns’ clustering method and distance: average and correlation, respectively.

## 3. Results

### 3.1. BMSCs Phenotypic Characterization

**Figure 1:**
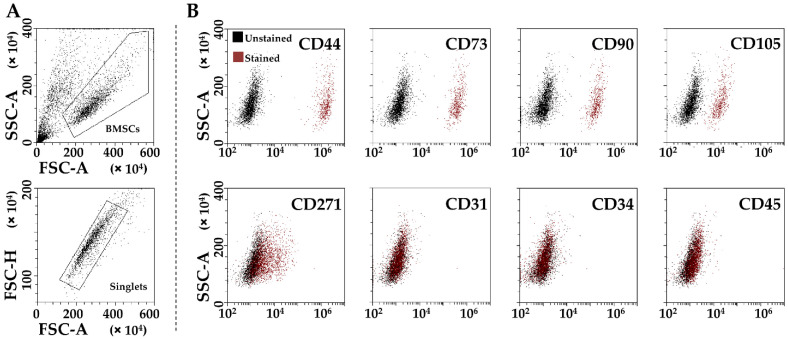
Flow cytometry analysis of BMSCs. (**A**), Identification of single cells by exclusion of debris (upper panel) and aggregates (lower panel). (**B**), staining for general mesenchymal (CD44, CD73, CD90 and CD105, positive), BMSC-specific (CD271, positive) and hemato-endothelial markers (CD31, CD34 and CD45, negative), confirming BMSCs identity. Representative plots are shown.

BMSCs were positive for mesenchymal (CD44/73/90/105) markers and negative for hemato-endothelial (CD31/34/45) ones (Figure 1A,B). CD271, considered a marker present in adult MSCs, was also detected at 17% ± 9, in agreement with previous findings [19].

### 3.2. BMSCs Secreted Factors

**Figure 2:**
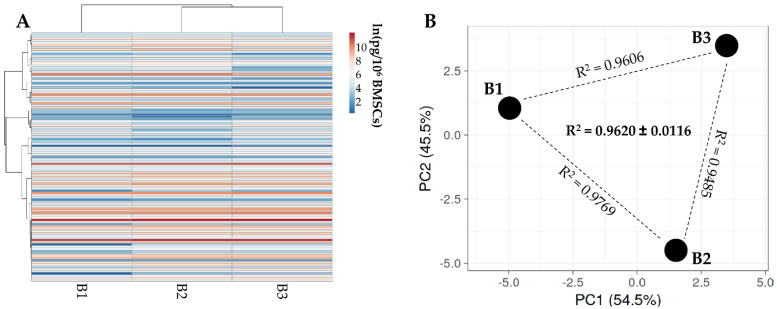
Comparison of secreted factor profiles between BMSCs under study. (**A**), heat map of hierarchical clustering analysis of the ln(x)-transformed pg/million BMSCs values of detected factors with sample clustering tree at the top. Absolute expression levels are indicated by the color scale: blue shades = low expression levels and red shades = high expression levels. (**B**) principal component analysis of the ln(x)-transformed pg/million BMSCs values of detected factors. X and Y axis show principal component 1 and principal component 2 that explain 54.5% and 45.5% of the total variance.

Out of 200 molecules, including inflammatory mediators and growth factors, chemokines, receptors and cytokines, 111 were present in all donors (Table 1). Hierarchical clustering showed higher similarity for BMSC 1 and 2 (Figure 2A), although within a pattern of overall conserved distance between donors in both PCA (Figure 2B) and correlation analysis (mean R^2^ of 0.96 ± 0.01). Thus, the average value was calculated to provide a guide to the level of each factor (Table 1). In 48 h, only insulin-like growth-factor-binding proteins-4 and -3 were secreted with an average amount superior to 100 ng per million BMSCs (IGFBP4, mean 132 ± 26; IGFBP3, mean 105 ± 16). Six other factors were detected between 10 and 100 ng: TIMP2 (41 ± 4), TGFB1 (20 ± 7), IFNL1 (18 ± 1), SERPINE1 (18 ± 2), TIMP1 (17 ± 3) and IGFBP6 (11 ± 1). It was also found that a further 24 factors had values ranging between 1 ng and 10 ng; the remaining 79 factors were below 1 ng.

**Figure 3:**
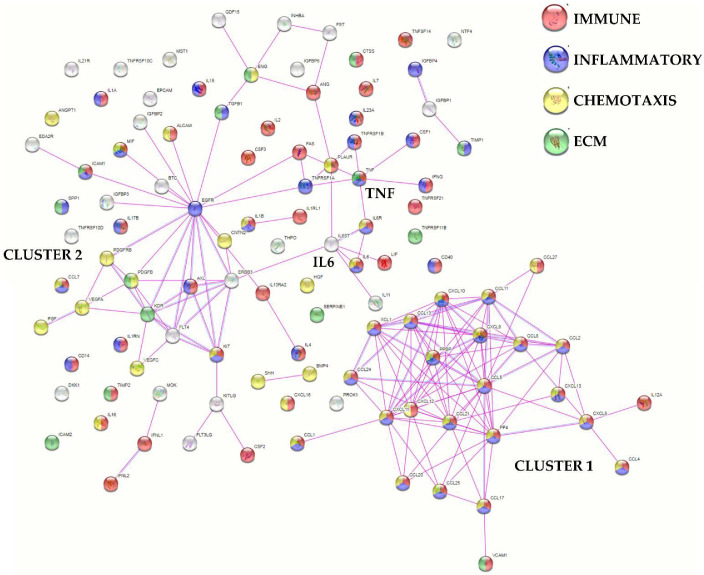
Functional association network for identified secreted factors. Protein–protein interaction levels for 111 proteins of the BMSCs secretome were mined using the online tool STRING. The blue connections are for proteins with known interactions based on curated databases; violet connections for proteins with experimentally determined interactions. Colorless nodes for proteins not related to the GO terms: immune, inflammatory, chemotaxis and ECM in the STRING database v 11.5. Empty nodes, proteins of unknown 3D structure; filled nodes, known or predicted 3D structure. Immune, inflammatory, chemotaxis and ECM-related GO terms are shown.

**Figure 4:**
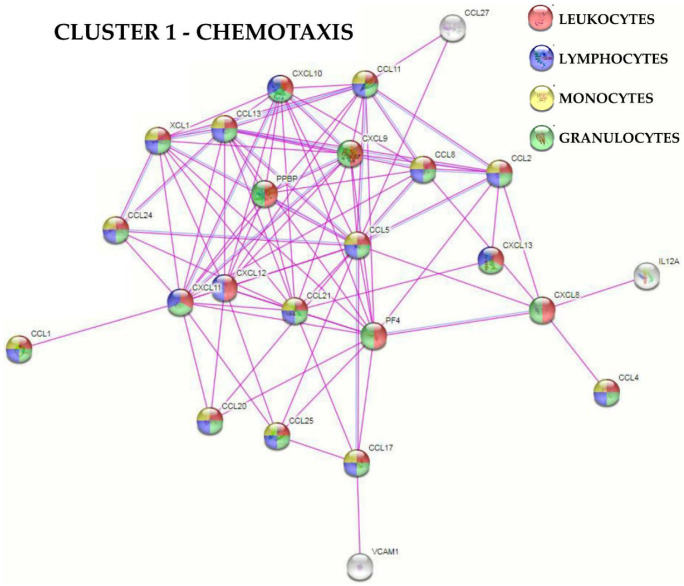
Functional association network for Cluster 1 secreted factors related to the GO term “chemotaxis”. Using the online tool STRING, protein–protein interaction levels for 24 proteins of the BMSCs Cluster 1 related to the GO term “chemotaxis” for leukocytes, lymphocytes, monocytes and granulocytes were mined. The different colors represent the immune cell type the “chemotaxis” term is associated with. The blue connections are for proteins with known interactions based on curated databases; violet connections for proteins with experimentally determined interactions. Colorless nodes for proteins not related to the GO terms: leukocytes chemotaxis, lymphocytes chemotaxis, monocytes chemotaxis and granulocytes chemotaxis in the STRING database v 11.5. Empty nodes, proteins of unknown 3D structure; filled nodes, known or predicted 3D structure.

Functional protein association network analysis based on experimental and database-annotated interactions allowed for the definition of two main clusters (Figure 3). The first cluster was tighter, composed of 24 factors, mainly involved in immune (gene ontology GO:0006955, Supplementary Table S1A for all analyzed factors) and inflammatory (GO:0006954) responses and chemotaxis (GO:0006935). The second cluster was looser, centered on EGFR with seven players defined by the GO term chemotaxis. Connected to this group, two smaller clusters emerged, one centered on IL6 and its receptor subunits (IL6R and IL6ST), while the other centered on TNF and its receptors (TNFRSF1A and B). Eventually, 14 molecules were related to extracellular matrix organization (GO:0030198), without the definition of a specific cluster (Figure 3). Dissecting further the GO term chemotaxis, several terms related to immune cells appeared. These were mainly associated with cluster 1 (Figure 4), which included leukocytes (30 overall factors, GO:0030595) and their subtypes: granulocytes (23, GO:0071621), lymphocytes (19, GO:0048247) and monocytes (17, GO:0002548) (Supplementary Figure S1 and Supplementary Table S1B for all analyzed factors). Interestingly, the leukocyte activation term was defined by several players (31, GO:0045321) without the identification of a specific cluster (Supplementary Figure S2 and Supplementary Table S1C for all analyzed factors). In this frame, lymphocytes (18, GO:0046649) and the subcategory T cells (12, GO:0042110) were the most present terms, followed by neutrophils (10, GO:0042119) and macrophages (4, GO:0042116).

### 3.3. Characterization of BMSC-EVs

**Figure 5:**
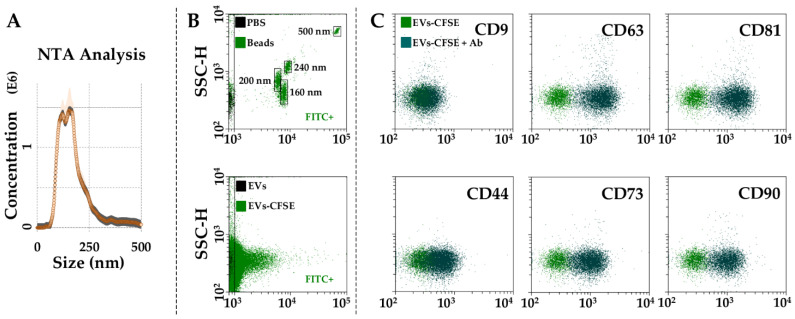
Characterization of BMSC-EVs. (**A**), EVs size analysis from NTA data. (**B**), flow cytometer was first calibrated to score FITC-fluorescent particles of nanometer scale (upper panel, starting from 160 nm). EVs were then CFSE stained to allow their identification and gating in the FITC channel (lower panel). (**C**), after gating, CFSE^+^ EVs showed positive staining for CD63 and CD81 extracellular vesicle defining molecules and CD44, CD73 and CD90 MSC markers. CD9, another EV postulated marker, was barely detectable. Representative cytograms are presented.

BMSCs released around 650 EVs per cell in 48 h. The mean size calculated using NTA technology resulted to be 138 nm ± 7 with 73% ± 1 of particles being below 200 nm (Figure 5A). Dimensional analysis was confirmed by flow cytometry through direct comparison, with FITC-fluorescent nanobeads showing 77% ± 1 of EVs below 200 nm (Figure 5B). Particles resulted positive for the EV markers CD63 (93% ± 1) and CD81 (91% ± 2). The particles were almost negative for CD9 (5% ± 1, Figure 5C) as already demonstrated for BMSC-EVs [19] and EVs from other MSC types, adipose- [20] or amniotic-membrane-derived [21]. With respect to MSC markers, CD44 staining gave a 46% ± 5 positivity, although the complete shift of the population suggests a homogeneous staining of all EVs. CD73 and CD90 were strongly positive, too (81% ± 3 and 83% ± 1, respectively, Figure 5C).

### 3.4. Identification of BMSC EV-miRNAs

**Figure 6:**
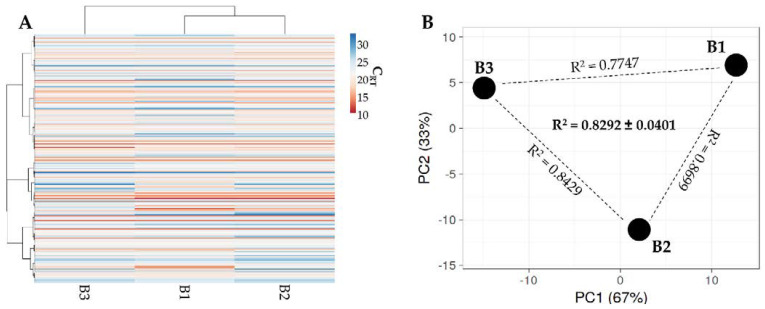
Comparison of EV-miRNA expression profiles between BMSCs under study. (**A**) heat map of hierarchical clustering analysis of the normalized C_RT_ values of detected miRNAs with sample clustering tree at the top. Absolute expression levels are indicated by the color scale: blue shades = low expression levels (high C_RT_ values) and red shades = high expression levels (low C_RT_ values). (**B**) Principal component analysis of the normalized C_RT_ values of detected miRNAs. X and Y axes show principal component 1 and principal component 2 that explain 67% and 33% of the total variance.

Out of 754 molecules, 201 miRNAs were detected in all samples (Supplementary Table S2). Hierarchical clustering was able to show higher similarity for BMSC 1 and 2 (Figure 6A), although the same pattern of overall conservation already observed for released factors emerged, characterized by a preserved distance between donors in both PCA (Figure 6B) and correlation analysis (mean R^2^ of 0.83 ± 0.04). Thus, as for released factors, an average miRNA C_RT_ value was calculated (Supplementary Table S2). Furthermore, since in MSC-EVs no more than one miRNA copy per EV is present [22], and no fewer than 100 EVs are needed to transfer one miRNA copy to a target cell [23], only those within the first quartile of expression were selected. This resulted in a list of 53 miRNAs, covering 97.2% of the detected genetic message (Table 2). The results show that the most represented miRNAs were hsa-mir-518f-3p (25.3% of the total weight), followed by hsa-miR-24-3p (11.5%) and hsa-miR-222-3p (7.7%). The miRNAs hsa-miR-720, hsa-miR-520e-3p and hsa-mir-193a-5p (all around 0.14%) were found at the bottom of the quartile. The miRNAs hsa-miR-720, as well as hsa-miR-1274A/B, were not considered further, as these are likely to be a fragment of a tRNA [24]. By sifting experimentally validated miRNA–mRNA interactions (Supplementary Table S3), 1152 univocal targets were identified (Supplementary Table S4). Gene ontology analysis of identified targets vs. the whole genome showed that the first ten enriched processes were related to biological, cellular and metabolic processes without a clear definition of regulated pathways (Table 3), preventing the definition of a disease-tailored prediction of efficacy. This is also emphasized by the transcriptional pattern in pathological tissues or cell types that may greatly diverge from the whole genome, further reducing the weight of broad bioinformatics analysis “vs. the whole genome”.

### 3.5. Target and Effect Prediction of BMSC EV-miRNAs on OA-Related Cell Types

To obtain a disease-framed influence of first quartile EV-miRNAs, initially identified univocal targets were compared with factors reported to be involved in OA at different levels, as well as factors expressed by at least 1% of resident chondrocytes, type B synoviocytes and immune cells [25]. EV-miRNAs in the first quartile targeted 32 molecules (Table 4), including 7 cytokines, 13 growth factors and 12 players related to proteolytic activities involved in extracellular matrix (ECM) homeostasis. Of note, all targeted cytokines are involved with inflammation and cartilage erosion, including the two most studied OA-driving molecules interleukin 1β (IL1B) and tumor necrosis factor α (TNF). Moreover, if also including cytokines expressed by a very limited percentage of synoviocytes and macrophages (<1%), interferon γ (IFNG) also emerged as mainly targeted by hsa-miR-24-3p (11.53%). For growth factors, several (10 out of 13) are endorsed with an OA-promoting capacity, especially related to ECM degradation and inflammation. In addition, hepatocyte growth factor (HGF) and connective tissue growth factor (CTGF) promote osteophyte formation, while transforming growth factor β (TGFB1) and KIT ligand (KITLG) promote synovial fibrosis and hyperplasia, making EV-miRNAs possible regulators of all OA-affected tissues. Eventually, 10 out of 13 protease-related factors are direct ECM-degrading enzymes. This includes the most studied matrix metalloproteinase (MMP)1, 9 and 13, with activated protein C (APC) being an MMP activity promoter. Altogether, cartilage-destructive and pro-inflammatory targets largely overcome protective molecules. With respect to single miRNA contribution, hsa-miR-24-3p was found to be the most abundant player (11.53% of the total weight) targeting TGFB1 and MMP14. The next most abundant player was hsa-miR-222-3p (7.71%), which regulates the expression of two molecules with opposite roles, such as MMP1 and the tissue inhibitor of metalloproteinases (TIMP)3. Two other miRNAs with almost identical weight, hsa-193b-3p (6.88%) and hsa-miR-191-5p (6.39%), target OA-inducing mRNAs’ plasminogen activator urokinase (PLAU) and interleukin 1 α (IL1A), respectively. Notably, hsa-miR-125b-5p (1.01%) is the molecule regulating the larger number of targets (six), followed by hsa-miR-145-5p (0.96%, three), hsa-miR-16-5p (0.82%, three) and hsa-miR-29a-3p (0.76%, 3). Lastly, type B synoviocytes appeared as the preferential EV-miRNAs interactors, since almost all identified targets are expressed by these cells (32 out of 33), followed by HLA-DR^+^ cells (19), chondrocytes (17) and T cells (2).

The second step was to compare identified first-quartile miRNAs with those reported to be directly involved with OA at cartilage [26] and synovia [27] levels or macrophage polarization [28] and T cell activation [29] (Table 5). Regarding cartilage, nine protective and six destructive miRNAs were identified, with hsa-miR-145-5p having a dual role. In the first group, five miRNAs were found to have a weight > 1%, driven by hsa-miR-24-3p (11.53%), hsa-miR-222-3p (7.71%) and hsa-miR-193b-3p (6.88%), for a total weight of 31.13%. In the second group, no miRNAs with a weight > 1% emerged, for a total weight of 3.98%. Therefore, the protective vs. destructive ratio was 10.3-fold. Concerning synovia, the definition of OA-related miRNAs is still in its infancy. Our data revealed two players, hsa-miR-29a-3p (0.76%), which reduces OA-induced synovia remodeling, and hsa-miR-34a-5p (0.30%), which upregulates synovial inflammation. Regarding immune cells, four miRNAs were related to anti-inflammatory M2 and two to pro-inflammatory M1 macrophage polarization. In particular, M2 polarizing features were driven by hsa-miR-24-3p (11.53%) and hsa-miR-222-3p (7.71%), for an M2 vs. M1 ratio of 10.2 fold. Eventually, 8 miRNAs have an activating and 3 miRNAs a repressing function on T cells, for a repression vs. activation ratio of 2.8 fold. Again, hsa-miR-24-3p (11.53%) led the anti-activation properties of EVs together with hsa-miR-125b-5p (1.01%), while only-activating hsa-miR-214-3p have a weight > 1%. Thus, overall, protective and anti-inflammatory signals largely overcame OA-driving inputs.

## 4. Discussion

In this manuscript, soluble factors and EV-miRNAs’ fingerprint gave the molecular basis for the observed regenerative and anti-inflammatory properties of BMSCs in the frame of OA [30,31,32] and, as a consequence, paved the way for the use of purified secretome or EVs as a cell-free therapeutic approach in the treatment of osteoarthritis.

Soluble factors analysis confirmed that BMSCs secrete several leukocyte chemokines able to attract a wide array of immune cells, such as lymphocytes, monocytes and granulocytes (Supplementary Figure S1). This mechanism is part of the immunosuppressive BMSCs activity that, after chemoattraction, relies on the immune-inhibitory effects of an array of soluble mediators such as indoleamine 2, 3-dioxygenase (IDO) and nitric oxide (NO) that are most active on cells in close proximity. As an example, NO produced by BMSCs suppresses responsiveness [33] and inhibits proliferation in T cells [34]. Similar results are observed for IDO [35]. This is of relevance in OA, since pro-inflammatory T cells are among the major constituents of both synovial membranes [36,37] and synovial fluids infiltrates [37,38]. Moreover, IDO triggers monocyte differentiation into anti-inflammatory M2 macrophages [39] that in turn suppress T cell proliferation [40], thus amplifying the immunosuppressive effect generated by MSCs. This is again crucial, since in both synovial membranes and fluids the M1/M2 ratio is tipped towards M1 polarization, and these pro-inflammatory macrophages are among the most abundant immune cell types contributing to cartilage damage and bone alterations through the release of cytokines such as IL1B and TNF. Eventually, BMSCs may also act on neutrophils, the most abundant type of granulocytes found at high levels in OA synovial membranes and fluids [41]. BMSCs may suppress hydrogen peroxide production in activated neutrophils [42,43], thus limiting the intensity of a respiratory burst upon inflammatory stimulation. To date, the role of neutrophils in OA is still underestimated. Thus, BMSCs chemoattraction towards the different subsets of leukocytes is a crucial mechanism to directly interact with activated immune cells, abundant in OA tissues, and reduce their pro-inflammatory status. Consistently, among the most abundant (>1 ng/million cells) factors related to chemotaxis, we found vascular endothelial growth factor α (VEGFA, granulocytes), X-C motif chemokine ligand 1 (XCL1, lymphocytes/monocytes/granulocytes), C-X-C motif chemokine ligand 16 (CXCL16, lymphocytes), C-C Motif Chemokine Ligand 21 (CCL21, lymphocytes/monocytes/granulocytes), platelet factor 4 (PF4, granulocytes), CCL2 (lymphocytes/monocytes/granulocytes) and CXCL11 (lymphocytes/granulocytes).

Another important feature framed by soluble factors is their involvement with ECM, which, during the progression of OA, is actively remodeled under inflammatory conditions due to increased action of the matrix metalloproteinases (MMPs) in combination with a reduction in their inhibitors (TIMPs). In mice, TIMP2 reduction leads to accelerated OA [44], while in dogs, TIMP2 expression is decreased in OA synovial fluids [45] and cartilage [46]. Intriguingly, in BMSCs secretome, TIMP1 and 2 resulted among the most released factors (>10,000 pg/mL) (Table 1). In a similar range, serpin family E member 1 (SERPINE1) was found. This protein, found at reduced levels in OA cartilage, counteracts the activity of elevated urokinase/tissue-type plasminogen activators [47] and positively correlates with cartilage synthesis during pathophysiologic processes [48]. Importantly, although not directly related to ECM, the urokinase plasminogen activator surface receptor (PLAUR) found at >1000 pg/mL may contribute to reducing plasminogen activation in plasmin, which in turn activates MMPs [49]. The other ECM-related secreted factor with an average level >10,000 is TGFB1. TGFBs play critical roles in regulating chondrocyte differentiation from early to terminal stages, including condensation, proliferation, terminal differentiation and maintenance of articular chondrocytes [50]. TGFB supplementation can enhance cartilage repair and is therefore a potential therapeutic tool [51], considering also its pleiotropic effects on T cells by inhibiting TH1, TH2 and CTL differentiation and in concert with other factors promoting TH17 or pTreg cell differentiation [52]. Eventually, other players found at high levels (>10,000 pg/mL), such as insulin-like growth factor (IGF) binding proteins IGFBP3/4/6 and IGFBP2 (nearly 8000 pg/mL), are indirectly related to ECM. IGF-1 regulates cartilage repair by promoting cartilage anabolism and inhibiting cartilage catabolism and IGFBPs, by altering the bioavailability and function of IGFs, may deliver IGFs-independent signals for chondrocyte survival [53]. Intriguingly, increased IGFBP levels were reported in both the synovial fluid and articular cartilage from OA patients [54], although this mechanism is still to be clarified. Therefore, BMSCs secretome may orchestrate ECM homeostasis at different levels regulating both catabolic and anabolic pathways.

EV-miRNAs resulted in targeting both the majority of OA-driving factors and OA-related cell types. Several pro-inflammatory molecules, mainly secreted by synoviocytes and HLA-DR+ (including macrophages) cells, emerged. In this group fell IL1B, IL6 and TNF, together with IL1A, the cytokine with the strongest regulation, being a target of hsa-miR-191-5p (6.39% of the total EV genetic weight). This is consistent with the reported capacity of MSCs to both suppress immune response, by inhibiting production of inflammatory cytokines in immune cells, and attenuate inflammation in osteoarthritic joints [55]. With respect to growth factors, many identified molecules were related with cartilage homeostasis and OA progression, with TGFB1 and (VEGFA) being the two most regulated (18.52% and 8.04%, respectively). In particular, TGFB1 is targeted by one of the most abundant miRNAs, hsa-miR-24-3p (11.53%). Therefore, TGFB1 levels are regulated in a twofold manner after BMSCs or secretome administration. On one side, the molecule is directly added as soluble mediator, and on the other side, EV-miRNAs reduce its de novo production. This might be of crucial importance, since excess of TGFB1 can enhance cartilage repair but may also result in synovial fibrosis and osteophyte formation [51]. Interestingly, its downregulation at the cellular level has been proposed as a therapeutic option, in association with exogenous supplementation of TGFB1, to locally inhibit TGF-beta production, as EV-miRNAs can do. Regarding VEGFA, its targeting may be crucial in counteracting possible detrimental effects due to its presence as a soluble factor in the secretome. In fact, VEGFA excess plays a key role in controlling chondrocyte catabolism and angiogenesis as a crucial step for endochondral ossification of cartilage progenitors, which ultimately leads to progressive ECM breakdown [56]. This regulation on ECM homeostasis is also emphasized by the numerous metalloproteinases targeted by EV-miRNAs, such as MMPs and a disintegrin and metalloprotease/a disintegrin and metalloproteinase with thrombospondin motifs (ADAM/ADAMTS). In particular, MMP1 and 14 resulted to be highly regulated (8.67% and 12.49%) with the main contribution of hsa-miR-222-3p (7.71%) and hsa-miR-24-3p, respectively. Moreover, MMP2 was a preferential target (2.56%). In addition, two other ECM-related molecules as PLAU and plasminogen activator tissue type (PLAT) emerged. In particular, PLAU is strongly targeted due to hsa-miR-193b-3p (6.88%). This mechanism may act in combination with the presence of a PLAU receptor (PLAUR) as a free molecule in the secretome, therefore reducing both PLAU synthesis at the cellular level and PLAU bioavailability as a soluble mediator. In fact, it has been proposed that both PLAU/PLAUR-mediated cell surface proteolysis and/or PLAUR-mediated signaling may promote inflammatory joint disease, indicating that disruption of this key proteolytic/signaling system could provide a novel therapeutic strategy to limit OA [57].

EV-miRNAs, together with the direct targeting of OA-related molecules, may also act at a more general level on the different cell types usually involved in the pathology (Table 5). Intriguingly, with the exception of the synovial membrane that needs further studies to define miRNA’s role in the definition of its pathological state, the most abundant EV-miRNAs resulted to have protective and anti-inflammatory features. In detail, EV-miRNAs had a preponderance towards protection for cartilage (ratio of 10.3), anti-inflammatory phenotype for macrophages (M2 vs. M1 ratio of 10.2) and anti-activation for T cells (ratio of 3.0). This is again consistent with the amelioration of cartilage structure and reduction in inflammation observed after MSCs or MSC-based products in OA patients [58,59,60]. In particular, two miRNAs tipped the balance towards healing capacity, hsa-miR-24-3p and hsa-miR-222-3p. hsa-miR-24-3p levels were lower in OA patients, and IL1B decreased its abundance in chondrocytes in vitro [61]. Consistently, its overexpression led to an increase in cell viability, reduction in pro-inflammatory cytokines and ECM degradation. Moreover, hsa-miR-24-3p plays an important role in macrophage polarization [62,63]. Its overexpression decreased the production of M1 phenotype markers and increased the production of M2 markers. Conversely, its knockdown promoted M1 and diminished M2 macrophage polarization. Eventually, regarding T cells, hsa-miR-24-3p represses IFNG production in human activated CD4^+^ [64] and CD8^+^ [65] T cells. Of note, hsa-miR-24-3p found in tumor EVs inhibited T-cell proliferation and differentiation [66]. Regarding hsa-miR-222-3p, it resulted in reduced OA in patients [67] and OA chondrocytes [68], and its overexpression led to the suppression of apoptotic death and ECM degradation. In addition, hsa-miR-222-3p was reported to be an M2 macrophage inducer [69], since its overexpression in macrophages induced polarization of the M2 phenotype. Consistently, tumor EVs miR-222-3p was shown to be an effective regulator in the polarization of M2 macrophages [70]. Finally, although found at lower levels with respect to hsa-miR-24-3p and hsa-miR-222-3p, hsa-miR-193b-3p was also reported to have a protective effect on cartilage. Its expression was elevated in chondrogenic MSCs, while being significantly reduced in OA cartilage [71]. Moreover, overexpression of hsa-miR-193b-3p suppressed MMP19 mRNA and inhibited the IL1B-induced expression of MMP19 in vitro [72] and strongly enhanced in vivo cartilage formation in mice [71]. Thus, these three miRNAs may be envisioned as therapeutic molecules, and strategies to increase their amounts in EVs would greatly enhance the protective and anti-inflammatory potential of BMSCs and their secretome. In this perspective, future studies aimed at increasing the knowledge of miRNA roles in synovia could further sharpen the understanding of these therapeutic features, this compartment being the most responsive within the joint for the inflammatory response [71,73].

We are aware that this report has some limitations. First, the number of donors is small. Nevertheless, the high correlation between single donors for both secreted factors and miRNAs suggests that the analyzed patterns are shared and consistent. This is further demonstrated by 84% of proteins >1000 pg/mL and 78% of miRNAs within the first quartile of expression having all three single values falling into the selected expression range, respectively. Moreover, the comparison of miRNAs falling in the first quartile of this work with those recently published for BMSC-EVs obtained from a different bone marrow source (femoral channel) [74] showed a 64% overlap, with 7 of the top 10 most-expressed miRNAs of our work being found in the other list, including those we observed as potential disease modifiers such as has-miR-24-3p, has-miR-222-3p and has-miR-193b-3p, which lay in the top 10 positions of both lists. Due to this high homogeneity of molecular patterns, we preferred to describe the shared signals able to interact with diseased cell types at pleiotropic and molecular levels, being aware that differences in a few factors may differently modulate the overall message at the patient level. For such analysis, a higher number of donors will have to be screened in the future to identify specific modulated pathways in the frame of the “personalized medicine” concept that relies on the selection of appropriate and optimal therapies based on the context of a patient’s molecular or cellular analysis; in this case, the secretome composition for OA. Second, the array of soluble factors and miRNAs is also limited. We preferred to score molecules with a deep characterization and several data available, including many reports related to their role in OA. Future studies sifting a wider portfolio of factors and miRNAs will be needed to better frame secretome’s and EVs’ fingerprints. Furthermore, data here-reported are obtained from culturing cells in standard conditions, thus relying on a MEM-based medium and fetal bovine serum (FBS) as supplement, collecting the secretome in absence of serum to minimize its interference in protein and EVs detection and because the presence of animal contaminants would not allow the secretome to be used as therapeutics by regulatory entities. Moreover, there is growing evidence that cells may respond differently depending on the in vitro culturing conditions, e.g., inflammatory [75] or mechanical [76] stimuli that may enhance MSCs anti-inflammatory behavior, or in vivo environment, e.g., proximity with other tissues and cell types (chondrocytes, synoviocytes and immune cells) [77] or the synovial fluid [78,79] for OA. Therefore, the molecular data of secretome or EVs that were presented in this manuscript might be the first milestone to decipher their potential as cell-free approaches, while for the use of MSCs as therapeutics, the effect of an OA environment will be crucial to better frame shuttled signals. Eventually, to reduce effects due to gender, only female donors were selected. We opted for this choice since the majority of OA patients are women [80]; albeit, we are aware that male donors might result in slight differences to be unraveled in future research works.

## 5. Conclusions

EV-miRNAs and secreted factors account for the immunomodulatory and healing potential of BMSCs in the musculoskeletal field. Several molecules target both specific cytokines/chemokines and several cell types shaping OA initiation and progression. This molecular fingerprint gives ground for the use of BMSCs, and especially their secretome/EVs, as therapeutics and supports the promising results obtained in the first trials and therapeutic procedures using these innovative and cutting-edge regenerative products for joint diseases.

## Figures and Tables

**Table 1:** BMSCs secreted factors detected in all donors and ordered by abundance of the mean values.

		pg/Million BMSCs per 48 h					
Role	Factor	B1	B2	B3	Mean	SD	Function
GF	IGFBP4	96,122	154,302	146,144	132,189	25,720	Insulin-like growth factor-binding protein 4
GF	IGFBP3	83,291	108,773	121,569	104,545	15,910	Insulin-like growth factor-binding protein 3
INF	TIMP2	36,744	39,361	45,708	40,604	3764	Metalloproteinase inhibitor 2
GF	TGFB1	24,241	25,312	10,951	20,168	6532	Transforming growth factor beta-1
CHE	IFNL1	18,892	18,815	15,755	17,821	1461	Interferon lambda-1
CYT	SERPINE1	14,532	17,906	20,270	17,570	2354	Plasminogen activator inhibitor 1
INF	TIMP1	14,137	16,618	20,310	17,022	2536	Metalloproteinase inhibitor 1
GF	IGFBP6	9478	11,134	13,129	11,247	1493	Insulin-like growth factor-binding protein 6
GF	BMP4	12,217	9427	6622	9422	2284	Bone morphogenetic protein 4
GF	IGFBP2	4926	11,110	7451	7829	2539	Insulin-like growth factor-binding protein 2
GF	VEGFA	4498	6802	6811	6037	1088	Vascular endothelial growth factor A
REC	VCAM1	5052	6333	6116	5834	560	Vascular cell adhesion protein 1
CHE	MIF	4137	4918	5835	4963	694	Macrophage migration inhibitory factor
INF	TNFRSF1A	3763	4516	4897	4392	471	Tumor necrosis factor receptor superfamily member 1A
CHE	XCL1	3176	5103	3256	3845	890	Lymphotactin
CYT	INHBA	3536	4186	3812	3845	266	Inhibin beta A chain
CYT	ICAM2	3013	4715	3609	3779	705	Intercellular adhesion molecule 2
CHE	CCL27	2460	6090	2445	3665	1715	C-C motif chemokine 27
CHE	CXCL16	2945	3952	3942	3613	473	C-X-C motif chemokine 16
CYT	FST	1925	3169	3700	2931	744	Follistatin
CHE	MST1	2232	2598	2135	2322	200	Hepatocyte growth-factor-like protein
CHE	CCL21	934	2655	3282	2290	992	C-C motif chemokine 2
CYT	ANGPT1	1542	2114	2277	1978	315	Angiopoietin-1
REC	PLAUR	1301	2257	1872	1810	393	Urokinase plasminogen activator surface receptor
CYT	IL6ST	1135	2164	1532	1610	424	Interleukin-6 receptor subunit beta
CHE	PF4	3191	387	1071	1550	1194	Platelet factor 4
INF	CCL2	841	1368	1855	1355	414	C-C motif chemokine 2
CYT	CTSS	1051	1535	1461	1349	213	Cathepsin S
CYT	ANG	1204	1397	1364	1322	85	Angiogenin
REC	ALCAM	1034	1342	1390	1255	158	CD166 antigen
CHE	CXCL11	1443	1370	828	1214	274	C-X-C motif chemokine 11
INF	IL11	1611	711	1261	1194	371	Interleukin-11
CYT	IL23A	530	1172	1263	989	326	Interleukin-23 subunit alpha
CHE	CCL25	257	965	1495	906	507	C-C motif chemokine 25
CHE	SPP1	742	954	848	848	86	Osteopontin
CYT	IL13RA2	683	873	557	704	130	Interleukin-13 receptor subunit alpha-2
CHE	LIF	960	251	844	685	311	Leukemia inhibitory factor
INF	IL6	530	641	795	655	109	Interleukin-6
INF	IL1RN	439	807	695	647	154	Interleukin-1 receptor antagonist protein
GF	HGF	383	537	741	554	147	Hepatocyte growth factor
CYT	CED	386	587	663	545	117	Diaphyseal Dysplasia 1
REC	PDGFRB	162	647	805	538	274	Platelet-derived growth factor receptor beta
REC	CD14	361	385	457	401	41	Monocyte differentiation antigen CD14
GF	KDR	167	575	350	364	167	Vascular endothelial growth factor receptor 2
CHE	CXCL10	307	456	290	351	75	C-X-C motif chemokine 10
CHE	IFNL2	371	182	466	340	118	Interferon lambda-2
GF	GDF15	255	334	345	311	40	Growth/differentiation factor 15
INF	TNFRSF1B	263	328	330	307	31	Tumor necrosis factor receptor superfamily member 1B
CHE	TNFSF14	402	245	213	287	83	Tumor necrosis factor ligand superfamily member 14
CHE	CXCL12	248	244	360	284	54	C-X-C motif chemokine 12, splicing variant alpha
INF	IL6R	324	294	197	272	55	Interleukin-6 receptor subunit alpha
CYT	IL17B	189	513	56	253	192	Interleukin-17B
REC	IL21R	210	194	353	252	71	Interleukin-21 receptor
INF	ICAM1	200	265	268	244	31	Intercellular adhesion molecule 1
CHE	AXL	177	427	80	228	146	Tyrosine-protein kinase receptor UFO
INF	IL16	172	162	349	228	86	Pro-interleukin-16
INF	IL1A	107	261	285	218	79	Interleukin-1 alpha
INF	TNF	122	158	363	215	106	Tumor necrosis factor
CYT	DKK1	182	299	156	212	62	Dickkopf-related protein 1
INF	PDGFB	150	89	288	176	83	Platelet-derived growth factor subunit B
GF	NTF4	112	170	233	172	49	Neurotrophin-4
GF	EGFR	141	170	203	171	26	Epidermal growth factor receptor
CYT	CXCL12	146	219	146	170	34	C-X-C motif chemokine 12, splicing variant beta
INF	CXCL9	161	194	144	166	21	C-X-C motif chemokine 9
INF	IL7	147	144	139	144	3	Interleukin-7
GF	TNFRSF11B	127	121	178	142	26	Tumor necrosis factor receptor superfamily member 11B
CYT	THPO	106	269	9	128	107	Thrombopoietin
INF	CXCL8	86	99	180	122	42	Interleukin-8
INF	CCL5	87	120	141	116	22	C-C motif chemokine 5
REC	CNTN2	49	193	90	111	61	Contactin-2
INF	IL15	87	88	150	108	30	Interleukin-15
CHE	CCL7	137	42	112	97	40	C-C motif chemokine 7
CHE	BTC	85	89	109	94	11	Probetacellulin
CYT	VEGFC	105	110	61	92	22	Vascular endothelial growth factor C
INF	IL2	46	135	66	83	38	Interleukin-2
INF	IFNG	45	148	44	79	49	Interferon gamma
INF	CSF2	95	81	53	76	18	Granulocyte-macrophage colony-stimulating factor
GF	KIT	88	50	83	74	17	Mast/stem cell growth factor receptor Kit
INF	CCL1	88	78	49	72	17	C-C motif chemokine 1
CYT	TNFRSF10D	56	108	13	59	39	Tumor necrosis factor receptor superfamily member 10D
REC	ENG	52	80	45	59	15	Endoglin
INF	IL4	60	30	85	59	22	Interleukin-4
CYT	SHH	47	58	71	58	10	Sonic hedgehog protein
CYT	CD40	66	77	32	58	19	Tumor necrosis factor receptor superfamily member 5
GF	IGFBP1	54	80	35	56	19	Insulin-like growth-factor-binding protein 1
REC	FAS	51	63	47	54	7	Tumor necrosis factor receptor superfamily member 6
CHE	CCL20	22	66	64	51	20	C-C motif chemokine 20
CHE	CCL8	55	50	24	43	14	C-C motif chemokine 8
INF	CSF3	57	41	32	43	10	Granulocyte colony-stimulating factor
INF	CSF1	31	32	54	39	10	Macrophage colony-stimulating factor 1
CYT	EPCAM	30	35	36	34	3	Epithelial cell adhesion molecule
GF	KITLG	40	11	37	30	13	Kit ligand
GF	PGF	13	33	34	27	10	Placenta growth factor
REC	EDA2R	1	41	33	25	17	Tumor necrosis factor receptor superfamily member 27
INF	IL1B	8	26	21	19	8	Interleukin-1 beta
GF	PROK1	19	13	16	16	2	Prokineticin-1
CYT	IL1RL1	3	30	11	15	11	Interleukin-1 receptor-like 1
GF	FLT4	22	7	12	14	6	Vascular endothelial growth factor receptor 3
REC	TNFRSF21	16	18	5	13	6	Tumor necrosis factor receptor superfamily member 21
REC	MOK	1	16	21	13	9	MAPK/MAK/MRK overlapping kinase
INF	CCL24	15	2	19	12	7	C-C motif chemokine 24
REC	ERBB3	23	10	1	12	9	Receptor tyrosine-protein kinase erbB-3
INF	CCL11	8	4	19	11	6	Eotaxin
REC	TNFRSF10C	9	16	4	10	5	Tumor necrosis factor receptor superfamily member 10C
CHE	CCL13	10	6	11	9	2	C-C motif chemokine 13
CHE	CCL17	6	7	9	7	1	C-C motif chemokine 17
REC	FLT3LG	6	7	7	7	0	Fms-related tyrosine kinase 3 ligand
INF	CCL4	4	6	8	6	2	C-C motif chemokine 4
CHE	PPBP	7	5	5	6	1	Platelet basic protein
INF	CXCL13	4	2	3	3	1	C-X-C motif chemokine 13
INF	IL12A/B	1	1	3	2	1	Interleukin-12 subunit alpha

CHE = chemokine; CYT = cytokine; GF = growth factor; ING = inflammation; REC = receptor.

**Table 2:** First quartile EV-miRNAs.

	C_RT_	C_RT_	C_RT_	C_RT_	C_RT_			C_RT_	C_RT_	C_RT_	C_RT_	C_RT_	
miRBase ID	B1	B2	B3	Mean	SD	Weight %	miRBase ID	B1	B2	B3	Mean	SD	Weight %
hsa-miR-518f-3p	10.20	10.12	13.23	11.18	1.45	25.25552	hsa-miR-409-3p	16.97	17.12	16.73	16.94	0.16	0.46626
hsa-miR-24-3p	12.66	12.19	12.09	12.31	0.25	11.52898	hsa-miR-618	11.91	15.11	23.98	17.00	5.10	0.44726
hsa-miR-222-3p	13.16	13.01	12.51	12.89	0.28	7.70535	hsa-miR-106a-5p	17.15	16.86	17.43	17.15	0.23	0.40375
hsa-miR-574-3p	13.11	13.22	12.77	13.03	0.19	6.99275	hsa-miR-657	18.32	16.08	17.18	17.19	0.91	0.39180
hsa-miR-193b-3p	13.08	13.23	12.87	13.06	0.15	6.87580	hsa-miR-221-3p	18.56	17.13	16.95	17.55	0.72	0.30620
hsa-miR-191-5p	13.20	13.30	13.00	13.16	0.13	6.39020	hsa-miR-34a-5p	15.92	17.18	19.68	17.59	1.56	0.29686
hsa-miR-484	13.91	14.04	13.83	13.93	0.09	3.76555	hsa-miR-627-5p	16.27	16.58	20.00	17.62	1.69	0.29156
hsa-miR-1274B	13.99	13.97	14.23	14.06	0.12	3.42521	hsa-miR-302c-3p	14.25	20.79	17.81	17.62	2.67	0.29149
hsa-miR-197-3p	14.17	14.20	14.56	14.31	0.18	2.89092	hsa-miR-92a-3p	18.04	17.63	17.67	17.78	0.19	0.25999
hsa-miR-320a-3p	14.27	14.17	14.73	14.39	0.24	2.74067	hsa-miR-132-3p	18.16	17.62	17.87	17.88	0.22	0.24247
hsa-miR-662	17.80	16.69	12.09	15.53	2.47	1.24360	hsa-miR-205-5p	14.88	20.81	18.07	17.92	2.42	0.23693
hsa-miR-523-3p	14.57	14.89	17.56	15.67	1.34	1.12287	hsa-miR-483-5	17.43	18.43	18.00	17.95	0.41	0.23125
hsa-miR-214-3p	15.85	15.62	15.77	15.75	0.10	1.06599	hsa-miR-382-5p	17.24	18.16	18.65	18.02	0.58	0.22137
hsa-miR-125b-5p	16.24	15.54	15.69	15.82	0.30	1.01269	hsa-miR-199a-3p	18.76	17.68	17.65	18.03	0.52	0.21903
hsa-miR-145-5p	16.24	15.98	15.45	15.89	0.33	0.96495	hsa-miR-31-5p	18.23	18.15	17.96	18.12	0.11	0.20659
hsa-miR-19b-3p	15.93	16.01	15.94	15.96	0.04	0.92244	hsa-miR-138-5p	18.27	17.82	18.36	18.15	0.24	0.20206
hsa-miR-342-3p	16.10	16.05	15.77	15.98	0.15	0.91121	hsa-miR-20a-5p	18.45	18.08	18.03	18.19	0.19	0.19663
hsa-miR-99a-5p	16.16	15.85	16.19	16.07	0.15	0.85571	hsa-miR-376c-3p	18.24	18.27	18.44	18.31	0.09	0.18027
hsa-miR-16-5p	16.46	15.99	15.94	16.13	0.23	0.81990	hsa-miR-146b-5p	18.68	18.58	17.93	18.39	0.33	0.17046
hsa-miR-30c-5p	16.37	16.19	16.06	16.20	0.13	0.77693	hsa-miR-28-3p	18.77	18.23	18.20	18.40	0.26	0.16979
hsa-miR-21-5p	16.68	15.99	15.96	16.21	0.33	0.77407	hsa-miR-194-5p	17.13	20.51	18.07	18.57	1.42	0.15075
hsa-miR-29a-3p	16.27	16.14	16.33	16.25	0.08	0.75516	hsa-miR-186-5p	18.83	18.72	18.18	18.58	0.28	0.14995
hsa-miR-30b-5p	16.58	16.21	16.35	16.38	0.15	0.68770	hsa-miR-720	18.81	18.48	18.66	18.65	0.14	0.14235
hsa-let-7b-5p	17.41	16.26	16.00	16.56	0.61	0.60872	hsa-miR-520e-3p	15.35	17.71	23.08	18.72	3.23	0.13621
hsa-miR-17-5p	17.00	16.75	16.94	16.90	0.10	0.48147	hsa-miR-193a-5p	19.25	18.40	18.54	18.73	0.37	0.13502
hsa-miR-1274A	16.88	16.85	16.99	16.91	0.06	0.47782	Total						97.2

**Table 3:** Gene ontology analysis of miRNA targets vs. the whole genome; first ten enriched biological processes.

GO	Biological Process	Count in Network	FDR
GO:0048518	Positive regulation of biological process	826 of 6112	2.85 × 10^−165^
GO:0048522	Positive regulation of cellular process	791 of 5579	4.50 × 10^−165^
GO:0048519	Negative regulation of biological process	762 of 5389	6.31 × 10^−154^
GO:0048523	Negative regulation of cellular process	720 of 4874	7.11 × 10^−150^
GO:0009893	Positive regulation of metabolic process	642 of 3893	3.33 × 10^−147^
GO:0010604	Positive regulation of macromolecule metabolic process	611 of 3600	1.93 × 10^−142^
GO:0031325	Positive regulation of cellular metabolic process	587 of 3413	5.69 × 10^−137^
GO:0051173	Positive regulation of nitrogen compound metabolic process	567 of 3239	1.25 × 10^−133^
GO:0031323	Regulation of cellular metabolic process	781 of 6239	1.77 × 10^−130^
GO:0019222	Regulation of metabolic process	823 of 6948	1.27 × 10^−129^

FDR = false discovery rate; GO = gene ontology classification.

**Table 4:** OA-related factors targeted by EV-miRNAs.

	Expressing Cell (>1%) *				Weight%	Main EV-miRNA (%)	Function
	C	S	H	T			
**CYTOKINES**							
TNF		X	X		1.49	hsa-miR-125b-5p (1.01)	Pro-inflammatory
IL6		X	X		0.17	hsa-miR-146b-5p (0.17)	Pro-inflammatory
IL1B		X	X		0.77	hsa-miR-21-5p (0.77)	Pro-inflammatory
IL1A		X	X		6.39	hsa-miR-191-5p (6.39)	Pro-inflammatory
CXCL12		X	X		0.52	hsa-miR-221-3p (0.31)	Articular cartilage matrix degeneration
CCL5		X	X	X	1.07	hsa-miR-214-3p (1.07)	Cartilage erosion
IL11	X	X	X		0.78	hsa-miR-30c-5p (0.78)	Pro-inflammatory
**GROWTH FACTORS**							
TGFB1	X	X	X	X	18.52	hsa-miR-24-3p (11.53)	Cartilage homeostasis, chondrocytes hypertrophy
IGF1		X	X		0.98	hsa-miR-29a-3p (0.76)	Promotes chondrocyte anabolic activity
FGF2	X	X			0.97	hsa-miR-16-5p (0.82)	Promotes catabolic and anti-anabolic effects in OA joints
BMP2	X	X	X		0.88	hsa-miR-17-5p (0.48)	Promotes cartilage regeneration
VEGFA	X	X	X		8.04	hsa-miR-320a-3p (2.74)	Chondrocyte catabolism
HGF		X	X		1.04	hsa-miR-16-5p (0.82)	Cartilage homeostasis, osteophyte formation
ANGPT2		X	X		1.97	hsa-miR-125b-5p (1.01)	Abnormal angiogenesis in OA
CTGF	X	X	X		1.98	hsa-miR-145-5p (0.96)	Promotes osteophyte formation and ECM degradation
KITLG	X	X	X		2.74	hsa-miR-320a-3p (2.74)	Promotes synovial mast cell hyperplasia and inflammation
TGFB2	X	X	X		1.73	hsa-miR-145-5p (0.96)	Cartilage homeostasis, high levels during OA development
INHBB		X			0.30	hsa-miR-34a-5p (0.30)	TGFB superfamily, upregulated in OA
IGF2	X	X			1.01	hsa-miR-125b-5p (1.01)	Promotes cartilage matrix levels
BDNF		X			1.06	hsa-miR-16-5p (0.82)	Promotes joint pain and inflammation
**PROTEASES**							
ADAM12	X	X			0.76	hsa-miR-29a-3p (0.76)	Metalloproteinase involved in ECM degradation
ADAM17	X	X	X		0.96	hsa-miR-145-5p (0.96)	Metalloproteinase involved in ECM degradation
ADAMTS9		X			0.76	hsa-miR-29a-3p (0.76)	Metalloproteinase involved in ECM degradation
MMP1		X			8.67	hsa-miR-222-3p (7.71)	Metalloproteinase involved in ECM degradation
MMP2	X	X			2.56	hsa-miR-125b-5p (1.01)	Metalloproteinase involved in ECM degradation
MMP9		X	X		0.24	hsa-miR-132-3p (0.24)	Metalloproteinase involved in ECM degradation
MMP14	X	X			12.49	hsa-miR-24-3p (11.53)	Metalloproteinase involved in ECM degradation
PLAU		X	X		6.88	hsa-miR-193b-3p (6.88)	ECM-degrading enzyme
PLAT	X	X			0.77	hsa-miR-21-5p (0.77)	ECM-degrading enzyme
APC	X	X			1.41	hsa-miR-125b-5p (1.01)	Promotes MMP activity
TIMP2	X	X			0.60	hsa-miR-106a-5p (0.40)	MMP inhibitor
TIMP3	X	X			9.27	hsa-miR-222-3p (7.71)	MMP inhibitor

C = chondrocytes; S = synoviocytes; H = HLA^–^DR^+^ cells; T = T cells * [25].

**Table 5:** EV-miRNAs involved in OA-related cell types and mechanisms.

Cartilage	Weight%	Role
***Protective***		
hsa-miR-24-3p	11.52898	Regulates chondrocyte senescence
hsa-miR-222-3p	7.70535	Reduces cartilage degradation
hsa-miR-193b-3p	6.87580	Reduces inflammation
hsa-miR-320a-3p	2.74067	Increases chondrocyte viability
hsa-miR-125b-5p	1.01269	Prevents aggrecan loss
hsa-miR-17-5p	0.48147	Induces autophagy
hsa-miR-221-3p	0.30620	Prevents ECM degradation
hsa-miR-92a-3p	0.25999	Increases collagen deposition
hsa-miR-199a-3p	0.21903	Anti-catabolic
**TOTAL**	**31.13017**	
***Destructive***		
hsa-miR-16-5p	0.81990	Cartilage degradation
hsa-miR-21-5p	0.77407	Negatively regulates chondrogenesis
hsa-miR-30b-5p	0.68770	Pro-apoptotic, ECM degradation
hsa-miR-34a-5p	0.29686	Pro-apoptotic
hsa-miR-483-5p	0.23125	Chondrocyte hypertrophy, ECM degradation and cartilage angiogenesis
hsa-miR-138-5p	0.20206	Cartilage degradation
**TOTAL**	**3.01185**	
***Dual***		
hsa-miR-145-5p	0.96495	Regulates chondrocyte proliferation and fibrosis
**SYNOVIA**		
***Protective***		
hsa-miR-29a-3p	**0.75516**	Protects synovial remodeling
***Destructive***		
hsa-miR-34a-5p	**0.29686**	Synovial inflammation
**MACROPHAGE**		
***M1***		
hsa-miR-125b-5p	1.01269	Pro-M1
hsa-miR-145-5p	0.96495	Pro-M1
**TOTAL**	**1.97764**	
***M2***		
hsa-miR-24-3p	11.52898	Pro-M2, anti-M1
hsa-miR-222-3p	7.70535	Pro-M2
hsa-miR-34a-5p	0.29686	Pro-M2
hsa-let-7b-5p	0.60872	Pro-M2
**TOTAL**	**20.13992**	
**T CELL**		
***Pro-activation***		
hsa-miR-214-3p	1.06599	Reduces PTEN repressor
hsa-miR-19b-3p	0.92244	Reduces PTEN repressor
hsa-miR-21-5p	0.77407	Reduces PTEN repressor
hsa-let-7b-5p	0.60872	Targets IL10
hsa-miR-17-5p	0.48147	Reduces PTEN repressor and promotes IFNG
hsa-miR-106a-5p	0.40375	Targets IL10
hsa-miR-221-3p	0.3062	Downregulates PIK3R1
hsa-miR-132-3p	0.24247	Downregulates PIK3R1
**TOTAL**	**4.80510**	
***Anti-activation***		
hsa-miR-24-3p	11.52898	Represses IFNG
hsa-miR-125b-5p	1.01269	Targets key molecules for T cell activation
hsa-miR-342-3p	0.91121	Downregulated during activation
**TOTAL**	**13.45289**	
***Dual***		
hsa-miR-31-5p	0.20659	Upregulates IL2, downregulated with activation
hsa-miR-146b-5p	0.17046	Reduces TREF6 repressor, downregulated with activation
**TOTAL**	**0.37706**	

## Data Availability

The raw data presented in this study are openly available in OSF at https://osf.io/e4uzj/?view_only=3cf3946cb97841cd8684ae5baea12cd6, accessed on 8 April 2022.

## Supplementary Materials

The following supporting information can be downloaded at: www.mdpi.com/article/10.3390/cells11213501/s1, Figure S1. Functional association network for secreted factors associated to GO term “chemotaxis”. Colorless nodes are for proteins that are not related to the GO terms: leukocytes chemotaxis, lymphocytes chemotaxis, monocytes chemotaxis and granulocytes chemotaxis in the STRING database v 11.5; Figure S2. Functional association network for secreted factors associated to the GO term “activation”. Colorless nodes are for proteins that are not related to the GO terms: leukocytes activation, lymphocytes activation, macrophages activation and neutrophils activation in the STRING database v 11.5; Table S1. Protein lists for the most representative GO terms identified within scored secreted factors; Table S2. EV-miRNAs detected in BMSCs secretomes; Table S3. Experimentally validated targets for first-quartile EV-miRNAs; Table S4. Comprehensive list of targets of all first quartile EV-miRNAs.
