# Supplementary material for: Secreted Factors and Extracellular Vesicles Account for the Immunomodulatory and Tissue Regenerative Properties of Bone-Marrow-Derived Mesenchymal Stromal Cells for Osteoarthritis
Source: Cells. 2022 Nov 4;11(21):3501. doi: 10.3390/cells11213501 (PMC9658264; doi:10.3390/cells11213501)
Supplement: Supplementary file 1 [file cells-11-03501-s001.zip › Table S1_Cells.pdf]

Table S1: Protein lists for the most representative GO terms identified within scored secreted factors

Table S1A

- GO:0006955 Immune response 63
MIF,CCL17,CSF3,CCL2,CCL1,CCL13,IL2,IL23A,IFNG,IL4,IL1RL1,LIF,IL1RN,CCL21,CCL27,IL17B,TIMP2,IL1A,IL1B,IL7,ICAM1,CXCL13,KIT,CXCL16,VCAM1,PPBP,PF4,IL15,TNFRSF21,CSF2,AXL,CCL11,IL16,CD14,CXCL10,ALCAM,CXCL8,CXCL11,CSF1,IFNL1,IFNL2,ANG,PLAUR,FAS,CCL20,CXCL9,XCL1,IL6R,CTSS,IL13RA2,CD40,TNFRSF1B,CCL7,CCL25,CCL8,CXCL12,IL6,TNF,CCL24,MYD88,TNFSF14,CCL5,CCL4

- GO:0006954 Inflammatory response 46
TNFRSF1A,MIF,TIMP1,CCL17,TGFB1,CCL2,CCL1,CCL13,IL23A,IFNG,IL4,IL1RN,CCL21,IL17B,IL1A,IL1B,ICAM1,IGFBP4,EGFR,CXCL13,KIT,PPBP,PF4,IL15,AXL,CCL11,CD14,CXCL10,CXCL8,CXCL11,CSF1,CCL20,CXCL9,XCL1,IL6R,CD40,TNFRSF1B,CCL7,CCL25,CCL8,SPP1,IL6,TNF,CCL24,CCL5,CCL4

- GO:0006935 Chemotaxis 43
MIF,CCL17,HGF,CCL2,CCL1,CCL13,BMP4,CCL21,CCL27,PDGFRB,IL1B,CXCL13,KIT,CXCL16,PPBP,PF4,SHH,CCL11,IL16,CXCL10,ALCAM,CXCL8,CXCL11,PDGFB,CNTN2,PLAUR,CCL20,CXCL9,XCL1,IL6R,ENG,CCL7,CCL25,CCL8,CXCL12,IL6,CCL24,ANGPT1,PGF,CCL5,VEGFA,VEGFC,CCL4

- GO:0030198 Extracellular matrix organization 14
TIMP1,TGFB1,SERPINE1,TIMP2,KDR,ICAM1,VCAM1,TNFRSF11B,PDGFB,CTSS,ENG,SPP1,TNF,ICAM2

Table S1B

- GO:0030595 Leukocyte chemotaxis 30
CCL17,CCL2,CCL1,CCL13,CCL21,IL1B,CXCL13,KIT,CXCL16,PPBP,PF4,CCL11,IL16,CXCL10,CXCL8,CXCL11,PDGFB,CCL20,CXCL9,XCL1,IL6R,CCL7,CCL25,CCL8,CXCL12,IL6,CCL24,CCL5,VEGFA,CCL4

- GO:0071621 Granulocyte chemotaxis 23
CCL17,CCL2,CCL1,CCL13,CCL21,IL1B,CXCL13,PPBP,PF4,CCL11,CXCL10,CXCL8,CXCL11,CCL20,CXCL9,XCL1,CCL7,CCL25,CCL8,CCL24,CCL5,VEGFA,CCL4

- GO:0048247 Lymphocyte chemotaxis 19
CCL17,CCL2,CCL1,CCL13,CCL21,CXCL13,CXCL16,CCL11,CXCL10,CXCL11,CCL20,XCL1,CCL7,CCL25,CCL8,CXCL12,CCL24,CCL5,CCL4

- GO:0002548 Monocyte chemotaxis 17
CCL17,CCL2,CCL1,CCL13,CCL21,CCL11,PDGFB,CCL20,XCL1,IL6R,CCL7,CCL25,CCL8,IL6,CCL24,CCL5,CCL4

TABLE S1C

- GO:0045321 Leukocyte activation 31
MIF,IL2,IL23A,IFNG,IL4,CCL21,TIMP2,IL7,IL11,ICAM1,KIT,VCAM1,PPBP,IL15,CSF2,SHH,AXL,IL12A,CD14,CXCL8,CSF1,IL21R,PLAUR,CTSS,CD40,TNFRSF1B,IL6,TNF,TNFSF14,FLT3LG,CCL5

- GO:0046649 Lymphocyte activation 18
IL2,IL23A,IL4,CCL21,IL7,IL11,ICAM1,KIT,VCAM1,IL15,SHH,AXL,IL12A,IL21R,CD40,IL6,TNFSF14,FLT3LG

- GO:0042110 T cell activation 12
IL2,IL23A,IL4,CCL21,IL7,ICAM1,KIT,IL15,SHH,IL12A,IL6,TNFSF14

- GO:0042119 Neutrophil activation 10
MIF,TIMP2,PPBP,IL15,CD14,CXCL8,PLAUR,CTSS,TNFRSF1B,CCL5

- GO:0042116 Macrophage activation 4
IFNG,IL4,CSF2,TNF
