# Supplementary figures and images for: Secreted Factors and Extracellular Vesicles Account for the Immunomodulatory and Tissue Regenerative Properties of Bone-Marrow-Derived Mesenchymal Stromal Cells for Osteoarthritis
Source: Cells. 2022 Nov 4;11(21):3501. doi: 10.3390/cells11213501 (PMC9658264; doi:10.3390/cells11213501)

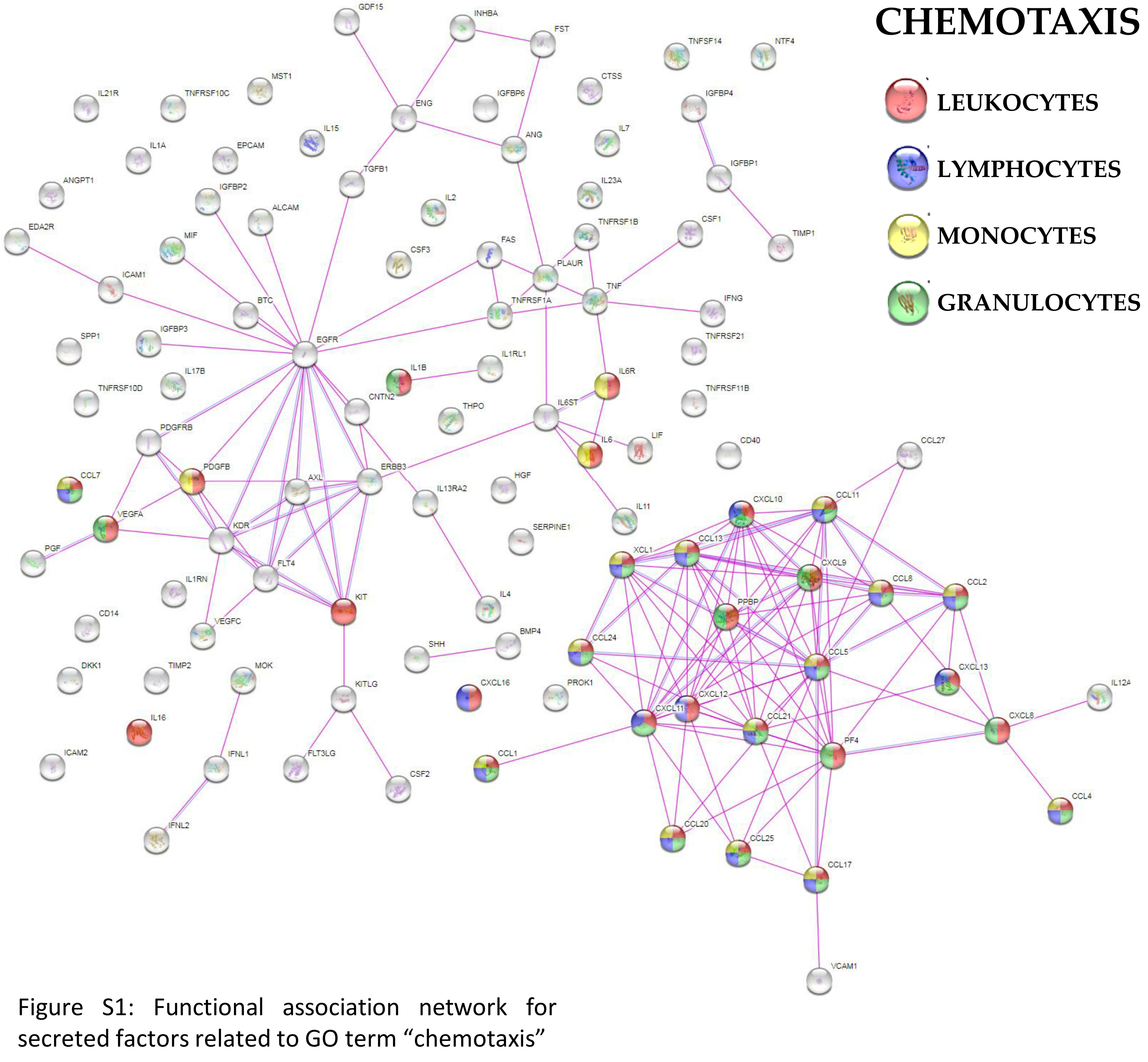

Supplement: Supplementary file 1 [file cells-11-03501-s001.zip › Figure S1_Cells.tif]

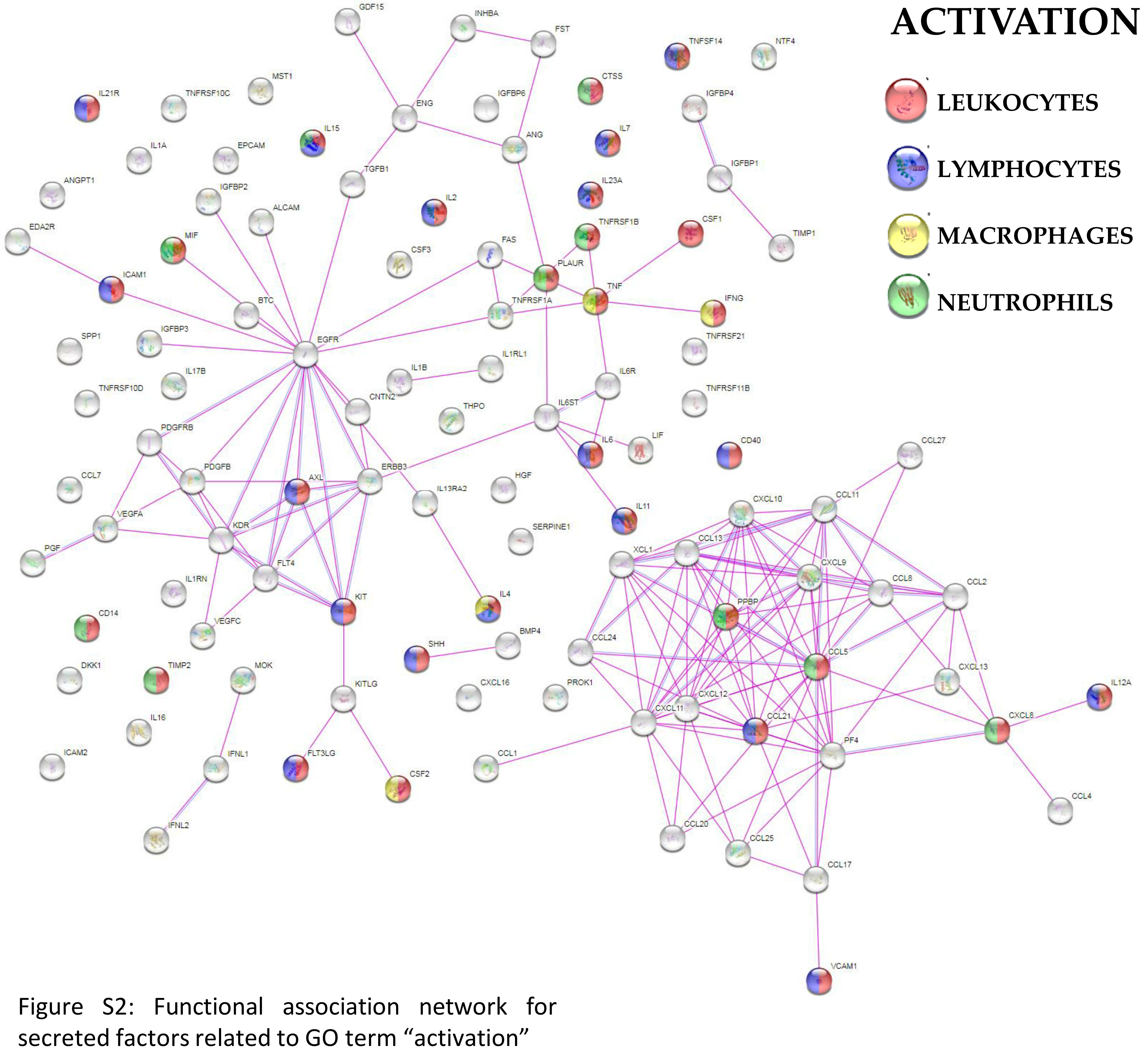

Supplement: Supplementary file 1 [file cells-11-03501-s001.zip › Figure S2_Cells.tif]

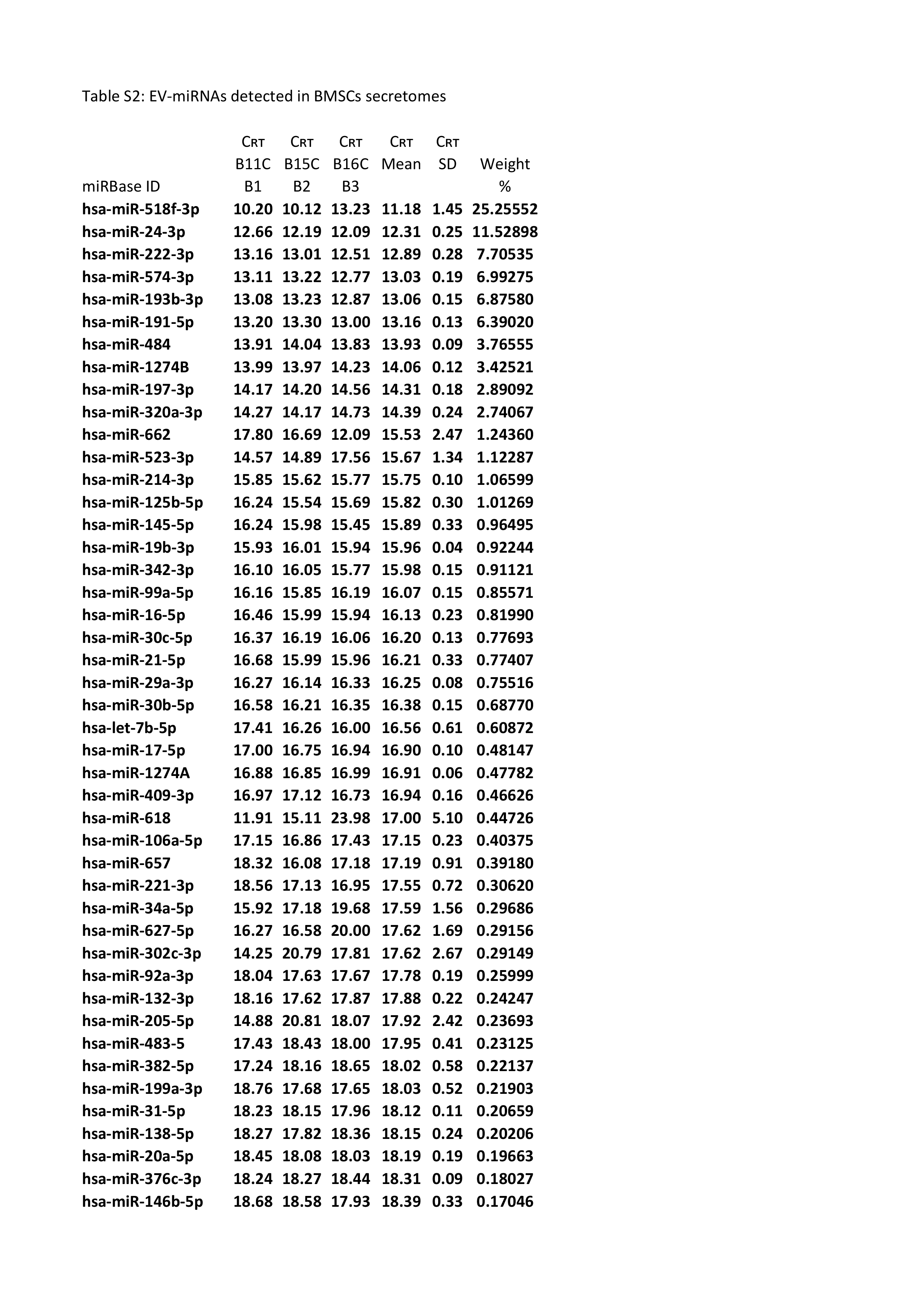

Supplement: Supplementary file 1 [file cells-11-03501-s001.zip › Table S2_Cells.tiff]

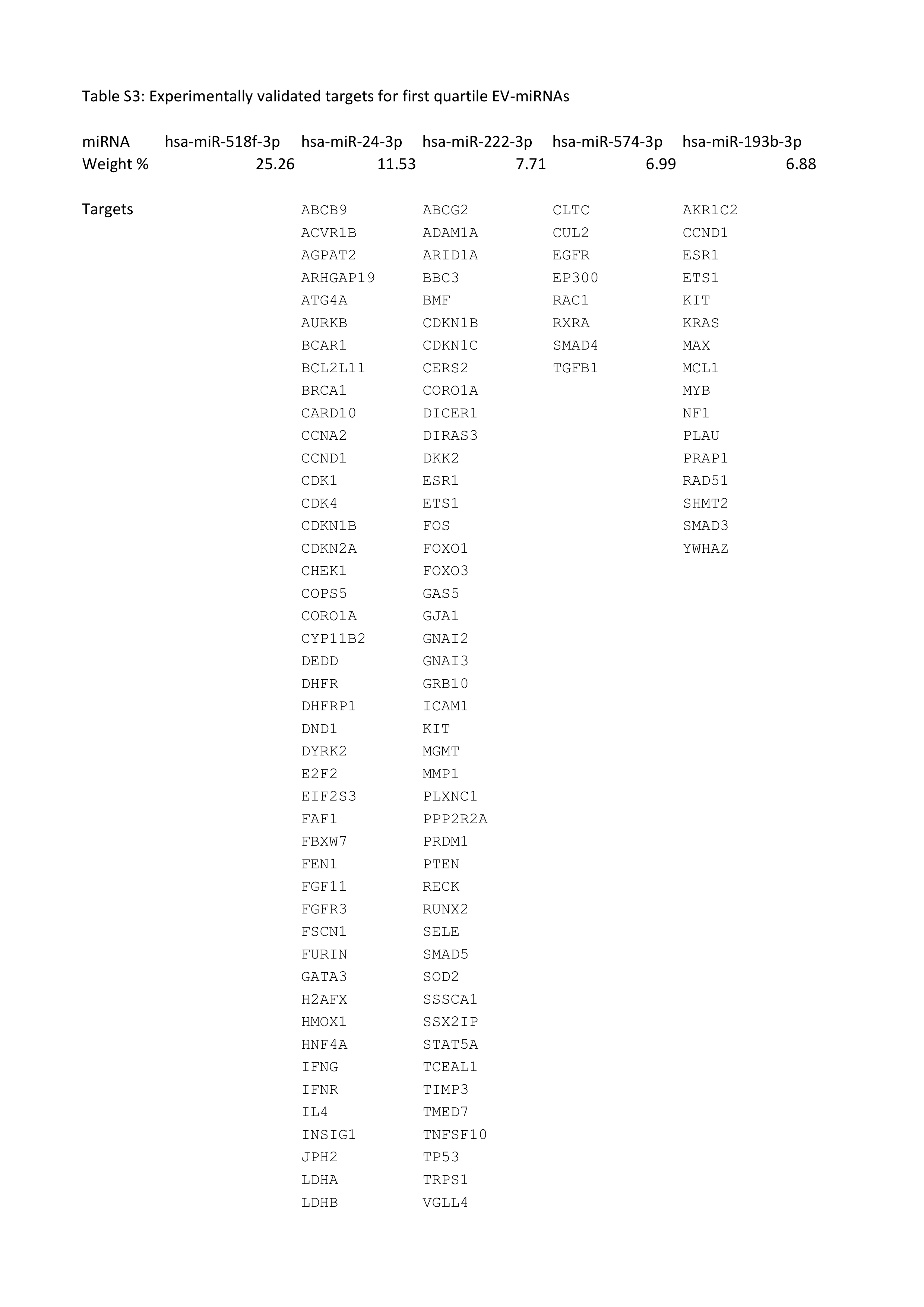

Supplement: Supplementary file 1 [file cells-11-03501-s001.zip › Table S3_Cells.tiff]

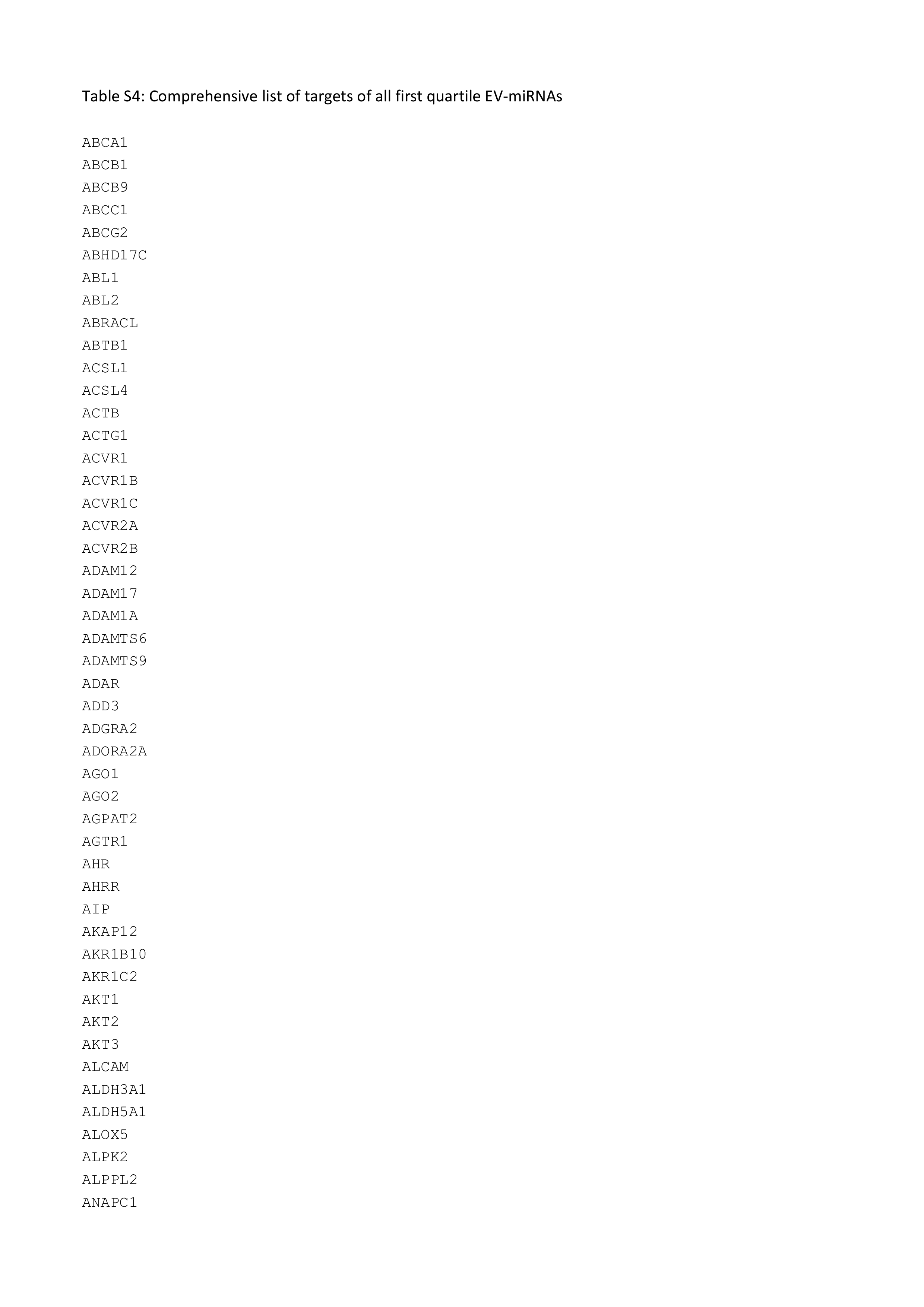

Supplement: Supplementary file 1 [file cells-11-03501-s001.zip › Table S4_Cells.tiff]
